# Authors’ response to “Evaluation of Treatments for HIV-Associated Kaposi Sarcoma in Africa”

**DOI:** 10.1186/s13027-021-00372-5

**Published:** 2021-05-05

**Authors:** Matthew E. Coldiron, Ana Gabriela Gutierrez Zamudio, Rolanda Manuel, Iza Ciglenecki, Laurence Toutous Trellu, Lucas Molfino

**Affiliations:** 1 Epicentre, 14-34 Avenue Jean Jaurès, 75019 Paris, France; 2 Médecins Sans Frontières, Maputo, Mozambique; 3 Ministry of Health, Maputo, Mozambique; 4 Médecins Sans Frontières, Geneva, Switzerland; 5 University Hospitals of Geneva, Geneva, Switzerland

**Keywords:** Kaposi sarcoma, Acquired Immunodeficiency Syndrome, Mozambique, AIDS-related opportunistic infections, Doxorubicin

We thank Drs Krown and Borok for the interest they have shown in our work and the opportunity they give us to draw attention to the extremely poor access to cancer treatment in resource-limited settings. Nonetheless, we feel it is important to clarify certain points.

Most importantly, safety follow-up of participants in our study was extensive. In addition to blood counts, serum creatinine and ALT were monitored before each PLD infusion, and PLD was held in case of abnormalities (as stated in the method section of the manuscript) [1]. Furthermore, the presence of AEs was assessed at each study visit, including at the regular PLD infusions. The type and severity of non-serious AE are described in the manuscript.

We agree with several of their concerns about the limitations of our single-site observational study, many of which we presented in the discussion of our manuscript. Our loss to follow-up (13 %) was indeed higher than in the clinical trial co-chaired by Drs Krown and Borok (though our follow up was 24 months vs. 48 weeks in the trial), but our study was not a clinical trial, and loss to follow up over the study period was much lower than in a historical cohort at the same center (36 %) [2, 3].

We used standard outcome measures used in multiple previous studies and believe that this was prudent given the single-site, observational nature of our study. We also point out that the duration of response was defined.

Importantly, we underscore that the study and all patient care provided at the study site (including HIV, TB and KS care) is a result of close collaboration and priority-setting between MSF and the Mozambican Ministry of Health.

Overall, we have not attempted to overstate our results and, as discussed in our manuscript, we believe that direct comparisons of PLD and paclitaxel are necessary. Yet, given the length of time it takes to set up and run such trials, we believe it is imperative to continue improving patient care now. The treatment gap for cancer patients in most of sub-Saharan Africa is a chasm. MSF is often a provider of last resort in these environments, with no viable chemotherapy alternatives available for KS or any other cancer.

Despite the limitations of our observational study, documenting our experience with PLD helped fill important information gaps for the Ministry of Health in Mozambique, led to changes in the national treatment protocol, and to financing for PLD by the Global Fund. PLD is a better solution for patients with advanced KS than the traditional ABV regimen, particularly in ambulatory settings, which is crucial for scale-up of cancer treatment. While waiting for results of direct comparisons between PLD and paclitaxel, which we welcome, this is a pragmatic step forward. Certainly, given its extensive use in high-income settings, PLD’s expansion into low-income settings like Mozambique is welcome. We hope other countries in resource-limited settings will follow Mozambique’s example and provide access to chemotherapy for patients with KS and other cancers.

## Data Availability

Not applicable

## Abbreviations

ALT: Alanine aminotransferase
PLD: Pegylated liposomal doxorubicin
AE: Adverse event
HIV: Human immunodeficiency virus
KS: Kaposi sarcoma
MSF: Médecins Sans Frontières (Doctors Without Borders)
ABV: Combination therapy with doxorubicin, bleomycin and vincristine
